# Assessing COVID-19 seroprevalence and vaccine uptake among women of reproductive-age in Zanzibar’s archipelago

**DOI:** 10.1371/journal.pgph.0003831

**Published:** 2025-07-07

**Authors:** Karim Manji, Ame Masemo Ame, Sayan Das, Ali Salim Ali, Navdeep Singh, Fahad Aftab, Arup Dutta, Saikat Deb

**Affiliations:** 1 Department of Paediatrics and Child Health, Muhimbili University of Health and Allied Sciences, Dar es Salaam, Tanzania; 2 Zanzibar Health Research Institute, Binguni, Zanzibar, Tanzania; 3 Centre for Public Health Kinetics, New Delhi, Delhi, India; 4 Center for Public Health Kinetics, Chake Chake, Tanzania; 5 Department of Interdisciplinary Studies, Swami Vivekanand Subharti University, Meerut, India; PLOS: Public Library of Science, UNITED STATES OF AMERICA

## Abstract

Recent events in public health globally have created a need for response by public health researchers in all disciplines to evaluate impact on health, morbidity and mortality of the COVID-19 pandemic that has engulfed the world after 100 years of previous influenza pandemic. Most of the immediate responses, globally; have focused on diagnosis and management of severe cases. However, for evolution of policies dealing with population health in the post COVID-19 era, community-based data for rates of morbidity, risk factors of illness and severity and outcomes of influenza like illness in Maternal, Neonatal and Child Health (MNCH) is needed to inform evidence-based policy. This pilot project aimed to collect preliminary data on seroprevalence from the two island populations of Tanzania. The proposed pilot, cross-sectional, facility-based study obtained a random sample of 1082 women of reproductive age visiting PHCUs from Unguja and Pemba. A total of 25 PHCUs were randomly selected among the 98 PHCUs. Venus blood sample was collected and a information related to recent history of covid-19 symptoms were collected in questionnaire from these women. The blood samples were processed for obtaining serum, which were tested for IgG and IgM antibodies at the PHl-CPHK laboratory in Pemba Island The overall IgG seroprevalence was 90.76% (95% CI: 89.01–92.51); Pemba: 92.91% (95% CI: 90.61–95.22), Unguja: 88.29% (95% CI: 85.38–91.20).There is minor non-significant difference in exposure between Unguja and Pemba in spite of large difference in tourist inflow between the islands. Severity profile and mortality experience is substantially lower in Zanzibar. The prevalence of Vaccination in community is Low and disinformation seems to be the main cause rather than reluctance. There is no significant relation with respect to symptomatology amongst seropositive along with their vaccination status.

## Introduction

In December 2019, an outbreak of pneumonia of unknown origin was reported among a group of patients linked to a seafood market in Wuhan, Hubei Province, China [1]. The causative agent was later identified as a novel strain of coronavirus, named severe acute respiratory syndrome coronavirus 2 (SARS-CoV-2), responsible for the disease known as COVID-19. By January 30, 2020, COVID-19 had been declared a public health emergency of international concern, yet many questions about the virus’s transmission and impact [2] remained unanswered.

As of September 7, 2022, there were 603,711,760 reported cases of COVID-19 worldwide, including 6,484,136 deaths. In the African region, 8,791,933 cumulative cases and 173,299 deaths were reported. Tanzania, which reported its first case in March 2020, accounted for 39,168 cumulative cases (0.45% of the region’s total) with an attack rate of 60.65 per 100,000 population and 845 cumulative deaths. Initially, the Tanzanian government implemented strict measures such as mask-wearing and social distancing. However, six months later, it declared the country free of COVID-19, lifted all restrictions, and ceased reporting new cases as of April 29, 2020. This stance persisted until early 2021, when a surge in high-profile deaths prompted the government to acknowledge the presence of COVID-19 but without reinstating preventive measures. Despite this policy shift, comprehensive data on COVID-19 in Tanzania remains sparse. Notably, the mortality rate in Africa is less than 1% of the global total, with Tanzania among the countries reporting the fewest cases and deaths [3,4]. Factors contributing to these low numbers include genetic factors, misdiagnosis, limited testing, and previous exposure to other infectious diseases, with misdiagnosis and under-reporting playing significant roles.

The reported numbers likely underestimate the true incidence of infections due to limited diagnostic testing [5,6], barriers to test accessibility [7], and asymptomatic cases [8]. As a result, the global prevalence of SARS-CoV-2 is underreported. The incubation period of the virus ranges from 2 to 14 days [5,6], and while most infected individuals exhibit mild symptoms, 10–20% may develop severe or critical illness [7]. Risk factors for severe disease include older age and comorbidities such as hypertension, diabetes, chronic obstructive pulmonary disease (COPD), and cardiovascular disease [8–10].

Reverse transcription polymerase chain reaction (RT-PCR) tests, which detect viral RNA, are the gold standard for COVID-19 diagnosis [11,12]. Although highly sensitive and specific [13,14], RT-PCR tests do not detect past infections or provide information on the host’s immune response [15]. Serological assays, which detect antibodies against SARS-CoV-2, complement nucleic acid testing by tracking the immune response and potentially informing prognosis [16,17] and immunity status. These tests identify antibodies indicative of past infection and can estimate the cumulative incidence of infection, aiding public health efforts [18,19].

Serological tests detect specific antibodies against SARS-CoV-2 antigens, with IgM produced in response to initial exposure and IgG following [20]. These tests are particularly useful for assessing immunity status over time, as antibodies against the S protein persist longer than those against the N protein [21]. Sero-epidemiological studies have become increasingly important in the context of vaccines and virus variants, helping to track infection spread, identify affected groups, and measure progress toward herd immunity [22–24]. However, few studies have thoroughly synthesized seroprevalence findings [25,26].

Tanzania initiated its COVID-19 vaccination campaign in July 2021, prioritizing health workers, elderly populations, and individuals with comorbidities. Vaccines were delivered through PHCU facilities and mobile outreach teams. Available vaccines in Zanzibar included Johnson & Johnson, Sinovac, Sinopharm, and Pfizer. However, coverage remained low, especially among reproductive-age women.

Data from two funded projects—a WHO COVID serosurvey study in Pemba and a COVID serosurvey in Zanzibar by the Amne Salim grant—allowed us to determine seropositivity among women of childbearing age in Zanzibar’s island populations. Vaccination data enabled comparisons between vaccinated and unvaccinated groups. This study provided the first community-based point prevalence of infections in East Africa by estimating IgM levels. Morbidity data further helped correlate seropositivity with symptomatic and asymptomatic cases among women.

This study aims to bridge the global knowledge gap by providing seroprevalence data from Zanzibar’s island populations, where epidemiological surveillance has been limited. Specifically, we examine seroprevalence and vaccination coverage among women of reproductive age, who represent a critical demographic for public health planning.

## Materials and methods

### Study area

Unguja: In Unguja, a stratified random sampling approach was used to select 25 PHCUs from seven districts using a fixed seed (2256). At least three PHCUs were selected from each district, with additional PHCUs added for larger districts. This ensured geographical representation (S1 File). At each selected PHCU, a pre-generated random list was used to recruit 20 eligible women of reproductive age who were visiting for postnatal care, vaccination, or child illness. Participants provided informed consent before enrolment.

Pemba: In Pemba, the study was embedded in a larger sero-surveillance cohort of 5000 women. For comparability, samples were drawn during April–July 2022 to match the Unguja sampling period. Trained staff visited households to recruit and consent participants, who were then scheduled for a study clinic visit where blood collection and symptom survey were conducted

### Ethical approval

All study protocols for were approved by ethical review committees of the World Health Organization (WHO) and appropriate local institutional review board Zanzibar Health Research Ethics Committee (ZAHREC). Participants provided written informed consent which included consent for drawing blood.

### Sample collection and morbidity surveillance

After consenting, detailed data collection preceded the blood collection. The data included clinical signs and symptoms of potential COVID like illness. The women were invited for a blood draw. From each participant 2–3 ml of venous blood was collected by venepuncture in BDBD Gel vacutainer tubes for obtaining serum. Blood was be stored in a cool box and transported to the Zanzibar Health Research Institute (ZAHRI) in case of Unguja and Public Health Laboratory of the Centre of Public Health Kinetics (PHL-CPHK) in case of Pemba) for centrifuging and separating serum... A clinic supervisor and a clinic officer were appointed who could oversee the execution of the project and its implementation in different clinics.

### Sample transport and analysis

In both the sites the sample vials were barcoded, and these barcode stickers were also inserted in the questionnaire as well as the cryovials which had the aliquoted serum. For samples collected in Unguja were transferred to prelabelled barcoded boxes and stored in -80 freezers till they were transported to the main serology lab in Pemba. Samples were transferred from Unguja to Pemba by Air in IATA certified Sample transfer boxes with dry ice. A detailed sample manifest was also included with each shipment. Samples from both the sites were analyzed for COVID-19 Specific antibodies using fully automated VIDAS Analyzer (Biomerieux, France). Both IgG and IgM antibody assays were performed using automated Enzyme linked Fluorescent Assay Based Technology. The assay principle combines a two-step sandwich enzyme immunoassay method with a final fluorescence detection (ELFA). Routine Calibration of the assays were performed using standard (Biomerieux, France) negative and positive controls for each Antibody Assay. Batch runs of IgG and IgM were performed separately for each of the samples against batch specific calibration curves. Quality control Checks were included with known in house controls (Serum samples obtained from both IgM, IgG Positive and negative individuals) collected during the epidemic. The VIDAS SARS-CoV-2 IgG and IgM ELFA assays target antibodies against the nucleocapsid and spike proteins. As such, these assays detect antibodies from both natural infection and vaccination. However, differentiation between the two sources of antibodies is not possible with the current assay and is noted as a limitation.

### Statistical analysis

COVID 19 virus specific antibodies IgG and IgM were semi quantified by VIDAS instrument (Biomeriux). Seropositivity was estimated separately for IgG and IgM. From the metadata collected via survey, the proportion of exposures were estimated and seropositivity tabulated against vaccination status. From the total number of seropositive women, the distribution of vaccinated and unvaccinated along with symptoms history was tabulated. For all kind of distribution, analysis was separately carried out for Pemba and Unguja.

Although the overall seroprevalence was high (>90%), logistic regression was retained for estimating associations due to its widespread interpretability and model convergence reliability in small sample subgroups. While Poisson regression with robust variance is often recommended in cases of high prevalence to avoid overestimation of relative risks, our primary aim was to assess direction and strength of association rather than estimate precise prevalence ratios. Furthermore, model diagnostics and sensitivity checks did not indicate major bias in odds ratio estimates.

### Definition of key variables

Socioeconomic status was calculated using an asset-based index via principal component analysis (PCA) and divided into wealth quintiles. Symptoms considered as COVID-like included fever, cough, sore throat, difficulty breathing, loss of smell or taste, and muscle pain, assessed within the two weeks prior to interview.

## Results

### Characteristics of the island population

A total of 578 and 504 were enrolled in the study from Pemba and Unguja respectively. Majority of women were less than 41years of age (Pemba: 493(~85%); Unguja: 475(~94%). Approximately 50% of women and their husbands in both the islands had primary or higher education. As seen in Table 1, the women had, private jobs such as farming, fishing and petty businesses. Likewise, most of the husbands had private sector jobs (Pemba: 42.91%; Unguja: 44.25%). Baseline socio–demographic data and assets inventory were used for classifying households into wealth quintiles. It is seen that the two island populations were similar to each other.

**Table 1:** Distribution of participants by age, occupations, and socio-economic factors.

Characteristics	Pemba (578)		Unguja (504)	
	n	%	n	%
*Women Age*				
<=30	236	40.83	340	67.46
31-40	257	44.46	135	26.79
41-50	85	14.71	29	5.75
*Women Education*				
No education	105	18.17	20	3.97
Primary	280	48.44	251	49.8
Secondary and higher	193	33.39	233	46.23
*Women Occupation*				
Government Sector	58	10.03	162	32.14
Private Sector	87	15.05	112	22.22
Farming and Fishing	116	20.07	34	6.75
Others	317	54.84	196	38.89
*Husband’s Education*				
No education	109	18.86	181	35.91
Primary	331	57.27	194	38.49
Secondary and higher	138	23.88	129	25.6
*Husband’s Occupation*				
Government Sector	84	14.53	56	11.11
Private Sector	248	42.91	223	44.25
Farming and Fishing	153	26.47	64	12.7
Others	93	16.09	161	31.94
*Wealth quintile*				
poor	147	25.40	101	20.00
poorest	111	19.20	101	20.00
middle	110	19.00	101	20.00
rich	108	18.70	101	20.00
richest	102	17.60	100	19.80

***Seroprevalence*** The overall IgG seropositivity was 90.76% (95% CI: 89.01–92.51). In Pemba, 92.91% of women were IgG positive (95% CI: 90.61–95.22), while in Unguja the IgG positivity was 88.29% (95% CI: 85.38–91.20). Among the 776 non-vaccinated women, 90.21% (95% CI: 88.27–92.15) tested positive for IgG antibodies. When stratified by island, IgG positivity among non-vaccinated women was 93.81% (95% CI: 91.18–96.43) in Pemba and 85.96% (95% CI: 82.30–89.62) in Unguja. IgM positivity was low overall (1.76%), with slightly higher prevalence in Pemba (2.94%) compared to Unguja (0.40%).Most of the IgM positive (3.51%) were above 40 years of age (Table 2).

**Table 2:** Overall distribution of seropositivity amongst the two islands of Zanzibar.

Testing	IGG			IGM		
	No of Samples Tested	Reactive	%	No of Samples Tested	Reactive	%
*Overall*	*1082*	*982*	*90.76* (89.01-92.51)	*1082*	*19*	*1.76 (0.97-2.55)*
Pemba	578	537	92.91 (90.61-95.22)	578	17	2.94 (1.52-4.35)
Unguja	504	445	88.29 (85.38-91.20)	504	2	0.4 (0.00-0.95)
*Among non-vaccinated*	*776*	*700*	*90.21 (88.27-92.15)*	*776*	*15*	*1.93 (0.98-2.89)*
Pemba	420	394	93.81 (91.18-96.43)	420	13	3.1 (1.45-4.74)
Unguja	356	306	85.96 (82.30-89.62)	356	2	0.56 (0.00-1.32)
By Age						
<=30	576	517	89.76 (87.21-92.31)	576	8	1.39 (0.43-2.35)
31-40	392	358	91.33 (88.52-94.13)	392	7	1.79 (0.49-3.09)
41-50	114	107	93.86 (89.91-97.81)	114	4	3.51 (0.12-6.89)

### Distribution of sero-prevalence with multiple morbidities and association with maternal characteristics

There were 558 women (51.6%) out of 1082 women who were asymptomatic in nature.

26.06% (282/1082) were vaccinated IgG positive and 64.7% (700/1082) non-vaccinated IgG positive. A total of 111 women had at least three COVID-like symptoms, including fever and 39 women has at least 3 symptoms excluding fever (Table 3). Out of the total 111 women who had at least three symptoms, 30 (7.09%) women were IgG positive as well as vaccinated and 10% women were IgG positive and non-vaccinated. A total of 558 women (51.6%) reported at least one COVID-like symptom in the two weeks preceding the survey.

**Table 3:** Overall distribution of seropositivity with morbidities amongst the two islands of Zanzibar.

Symptomatic/ Asymptomatic	N	IgG Negative		IgG Positive				IgM Negative		IgM Positive			
				Vaccinated		Non-Vaccinated				Vaccinated		Non-Vaccinated	
		N	%	N	%	N	%	N	%	N	%	N	%
*Total*	*1082*	*100*	*9.24*	*282*	*26.06*	*700*	*64.7*	1063	98.24	4	0.37	15	1.39
Asymptomatic	558	36	36	135	47.87	387	55.29	543	97.31	2	0.35	13	2.33
Symptomatic*	25	5	20	6	24	14	56	25	100.00	0	0.00	0	0

* Fever plus any COVID like symptoms.

Variables included in regression were chosen based on bivariate association (p < 0.2) and theoretical relevance. These included participant age, education, occupation, partner education, occupation, and wealth quintile.

### Vaccination uptake

**Fig 1:**
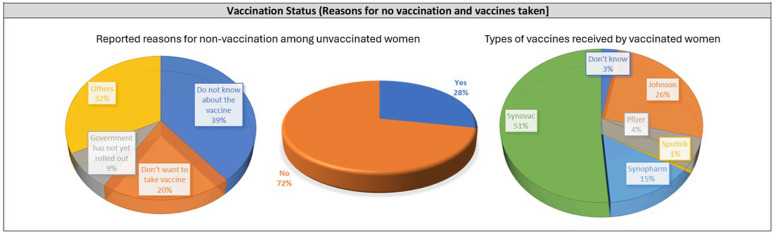
Vaccination details.

There were 776 non-vaccinated women (420 from Pemba and 356 from Unguja) (Fig 1). Lack of information was the main reason in Pemba (286/420, where 68.06% didn’t have any information about vaccines. In Unguja, 241(67.69%) out of 356 non vaccinated individual had multiple non-specific reasons of not getting vaccinated in Unguja (Fig 1). Synovac was found to be the most predominant vaccine to be used in Pemba and Johnson in Unguja. A detailed vaccination details by company and major reasons for non-vaccinations has been given in S1 Table.

## Discussion

The findings from this Zanzibar study offer a unique perspective to compare local data with global trends and explore the broader implications of COVID-19 on women of reproductive age. The research conducted in Pemba and Unguja highlights a notably high seropositivity rate, with Pemba exceeding Unguja. Among non-vaccinated women, Pemba’s IgG positivity reaches 93.81%, compared to Unguja’s 85.96%. These high seroprevalence rates are uncommon in global studies, where rates among women of reproductive age vary widely.

For instance, a systematic review of cytomegalovirus (CMV), which shares transmission pathways with SARS-CoV-2, found CMV IgG seroprevalence among women of reproductive age in Europe, Japan, Latin America, and North America ranged from 45.6-95.7%, 60.2%, 58.3-94.5%, and 24.6-81.0%, respectively [27]. These figures suggest that Pemba and Unguja’s seroprevalence rates are at the higher end, possibly due to unique geographical and socio-economic factors.

Demographic characteristics of the study population, such as most women being under 41 years old and about half having primary or higher education, align with global trends (Table 4). However, the study’s findings on occupational diversity and economic stratification are particularly noteworthy as they may influence health outcomes and access to healthcare services.

**Table 4:** Association of women characteristics with COVID 19 IgG positivity.

Characteristics	n (%)	Univariate			Multivariate		
		Odds ratio	95% CI	P	Odds ratio	95% CI	P
Women’s Age							
<=30	576 (46.77)	*Reference*					
>30	506 (53.23)	1.29	0.85 to 1.97	0.23	1.38	0.89 to 2.12	0.15
Women Education							
Yes	957 (11.55)	*Reference*					
No	125 (88.45)	0.78	0.43 to 1.42	0.42	0.76	0.40 to 1.45	0.4
Women’s Occupation							
Govt/private/self employed	419 (61.28)	0.94	0.62 to 1.44	0.78	0.77	0.49 to 1.21	0.25
Daily wage/ Farmer/ Other	663 (38.72)	*Reference*					
Husband’s Education							
Yes	792 (26.8)	*Reference*					
No	290 (73.2)	1.18	0.73 to 1.90	0.51	1.42	0.85 to 2.37	0.19
Husband’s Occupation							
Govt/private/self employed	550 (49.17)	*Reference*					
Daily wage/ Farmer/ Other	532 (50.83)	0.81	0.53 to 1.22	0.31	0.8	0.51 to 1.24	0.32
Wealth quintile							
Poor	460 (42.51)	*Reference*					
Middle	211 (19.5)	1.1	0.64 to 1.89	0.72	1.09	0.62 to 1.89	0.76
Rich	411 (37.99)	1.61	1.00 to 2.59	0.05	1.64	0.98 to 2.74	0.04

A significant finding is the association of seropositivity with maternal characteristics, particularly the educational status of husbands. The study indicates that women whose husbands had no education were twice as likely to be IgG positive. This correlation is not commonly reported in global studies and may reflect socio-cultural factors affecting health-seeking behavior and virus exposure in this population.

Vaccination uptake is a significant challenge in both Pemba and Unguja, with many women lacking information about vaccines. This situation mirrors many parts of Sub-Saharan Africa, where vaccine hesitancy and misinformation pose major obstacles. The preference for Sinovac in Pemba and Johnson & Johnson in Unguja also highlights the variations in vaccine availability and preferences across regions.

The high seroprevalence in the face of low vaccination uptake emphasizes the importance of targeted communication campaigns to address misinformation. This study highlights the utility of serosurveillance to detect silent transmission in low-testing settings, and the need for integrating such approaches into outbreak preparedness frameworks, particularly for vulnerable sub-populations.

The study confirms that COVID-19 exposure was present in Zanzibar (Unguja and Pemba). It quantified exposure, revealing that approximately 88.3% of women in Unguja and 92.9% of women in Pemba have been exposed to COVID-19, as indicated by IgG antibodies. Despite significant differences in tourist inflow, the exposure rates between Unguja and Pemba showed minor, non-significant differences. While COVID-19 exposure levels in Zanzibar are comparable to those in Asia and developed countries, the severity and mortality rates are significantly lower.

The low vaccination rates in the community are primarily due to misinformation rather than reluctance. There is no significant relationship between symptomatology among seropositive individuals and their vaccination status. This study is the first in East Africa and Tanzania to provide ongoing community-based point prevalence of infections by estimating IgM (approximately 2%). In the absence of formal data during the epidemic, this study offers the most representative community-based data on COVID-19 exposure and immunity in Zanzibar, providing valuable insights to guide policy and further research.

Our study has several limitations. First, selection bias may exist as Unguja participants were recruited from PHCUs, while Pemba women were drawn from a pre-existing cohort, potentially limiting representativeness. Second, symptom data and vaccination status were self-reported, introducing recall and reporting bias. Third, our regression model did not adjust for all potential confounders such as comorbidities or health-seeking behaviour. Finally, the serological assay used does not distinguish vaccine-induced from infection-induced antibodies, limiting interpretation.

## Conclusion

In conclusion, our study reveals that the population in Zanzibar has been exposed to COVID-19, indicating active transmission of the disease. This underscores the necessity of implementing vaccination strategies and enhancing surveillance as critical public health tools to protect vulnerable populations. The severity of COVID-19 in this population has been relatively mild compared to non-African populations. This could be due to the demographic structure, with a smaller proportion of the population being over 65 years of age, or potential prior cross-reactivity, as suggested by some findings in Africa and our study in Pemba. Further investigation is needed to determine the implications of these factors as COVID-19 becomes endemic.

Moreover, with the emergence of new, more mutated strains of the virus, it is crucial to assess the risk these variants pose to the population. Conducting neutralizing antibody studies using the bio-banked samples from this study could provide valuable insights. These studies would help evaluate the effectiveness of the immune response against new strains and inform future public health strategies.

## Supporting information

S1 Table Vaccination details and the differences in the uptake. (PDF)

S2 Table IgG and IgM Seropositivity Stratified by Key Variables. (PDF)

S1 File Randomized PHCU(s) in Zanzibar. (PDF)
