# Supplementary material for: Assessing COVID-19 seroprevalence and vaccine uptake among women of reproductive-age in Zanzibar’s archipelago
Source: PLOS Glob Public Health. 2025 Jul 7;5(7):e0003831. doi: 10.1371/journal.pgph.0003831 (PMC12233267; doi:10.1371/journal.pgph.0003831)
Supplement: S1 Table — (PDF) [file pgph.0003831.s001.pdf]

S1 Table: Vaccination details and the differences in the uptake			
**Vaccination Details**	**Pemba**	**Unguja**	**Total Vaccinated**
Vaccine Name			
Don't know	2	7	9
Johnson	0	80	80
Pfizer	2	9	11
Sputnik	0	2	2
Synopharm	46	1	47
Synovac	108	49	157
Total	158	148	306
Not Vaccinated reason			
Do not know about the vaccine	286	16	302
Don’t want to take vaccine	69	89	158
Government has not yet rolled out	56	10	66
Others	9	241	250
Total	420	356	776
