# Supplementary material for: Assessing COVID-19 seroprevalence and vaccine uptake among women of reproductive-age in Zanzibar’s archipelago
Source: PLOS Glob Public Health. 2025 Jul 7;5(7):e0003831. doi: 10.1371/journal.pgph.0003831 (PMC12233267; doi:10.1371/journal.pgph.0003831)
Supplement: S2 Table — (PDF) [file pgph.0003831.s002.pdf]

Supplementary Table 2: IgG and IgM Seropositivity Stratified by Key Variables

Variable	N	IgG Positive	IgG Positive (%)	IgM Positive	IgM Positive (%)
Age ≤30	576	517	89.76%	8	1.39%
Age 31–40	392	358	91.33%	7	1.79%
Age 41–50	114	107	93.86%	4	3.51%
Education: No	125	110	88.00%	3	2.40%
Education: Yes	957	872	91.14%	16	1.67%
Occupation: Govt/Private/Self	419	257	61.34%	4	0.95%
Occupation: Daily wage/Farmer/Other	663	444	66.97%	15	2.26%
Husband Education: No	290	254	87.59%	4	1.38%
Husband Education: Yes	792	728	91.92%	15	1.89%
Husband Occupation: Govt/Private/Self	550	465	84.55%	7	1.27%
Husband Occupation: Daily/Farmer/Other	532	517	97.18%	12	2.26%
Wealth: Poor	460	414	90.00%	11	2.39%
Wealth: Middle	211	191	90.52%	5	2.37%
Wealth: Rich	411	377	91.73%	3	0.73%
Island: Pemba	578	537	92.91%	17	2.94%
Island: Unguja	504	445	88.29%	2	0.40%
Vaccinated	306	282	92.16%	4	1.31%
Non-Vaccinated	776	700	90.21%	15	1.93%
