# Supplementary material for: Assessing COVID-19 seroprevalence and vaccine uptake among women of reproductive-age in Zanzibar’s archipelago
Source: PLOS Glob Public Health. 2025 Jul 7;5(7):e0003831. doi: 10.1371/journal.pgph.0003831 (PMC12233267; doi:10.1371/journal.pgph.0003831)
Supplement: S1 File — (PDF) [file pgph.0003831.s003.pdf]

**S1 File:** Randomized PHCU(s) in Zanzibar

Selection of PHCU is being done through stratified randomization by district using a seed value of 2256. Minimum of three PHCU from each of the 7 districts selected and for larger districts with a greater number of PHCU(s) (Kaskazini A, Kati, Magharibi B) we have selected 1 additional each from Kaskazini A and Magharibi B and 2 additional from Kati to have representational sample from each district.

Following are the selected PHCU(s) where the study was planned to be undertaken.

| **District** | **PHCU** | **Type_PHCU** |
|---|---|---|
| Kaskazini A | 6. Chaani Masingini | PHCUs |
| Kaskazini A | 3. Kijini | PHCUs |
| Kaskazini A | 8. Tazari... | PHCUs |
| Kaskazini A | 9. Mkokotoni | PHCUs |
| Kaskazini B | 4. Bumbwini Makoba | PHCUs |
| Kaskazini B | 1. Donge Vijibweni | PHCUs |
| Kaskazini B | 7. Kitope Mbaleni | PHCUs |
| Kati | 20. Mwera Pongwe | PHCUs |
| Kati | 16. Jendele | PHCUs |
| Kati | 12. Ndijani Baniani | PHCUs |
| Kati | 3. Bambi | PHCUs |
| Kati | 4. Umbuji | PHCUs |
| Kusini | 6. Bwejuu | PHCUs |
| Kusini | 1. Jambiani | PHCUs+ |
| Kusini | 1. Muungoni | PHCUs |
| Magharibi A | 1. Selem | PHCUs+ |
| Magharibi A | 7. Kianga | PHCUs |
| Magharibi A | 3. Chuini | PHCUs |
| Magharibi B | 2. Chukwani | PHCUs+ |
| Magharibi B | 3. Magogoni | PHCUs+ |
| Magharibi B | 2. K/ Samaki | PHCUs |
| Magharibi B | 1. Fuoni | PHCUs+ |
| Mjini | 3. Rahaleo | PHCUs+ |
| Mjini | 1. Mpendae | PHCUs+ |
| Mjini | 7. Sebleni | PHCUs |
